# Silver(I)-directed growth of metal-organic complex nanocrystals with bidentate ligands of hydroquinine anthraquinone-1,4-diyl diethers as linkers at the water-chloroform interface

**DOI:** 10.1186/1556-276X-9-488

**Published:** 2014-09-12

**Authors:** Ying Tang, Hui-Ting Wang, Meng Chen, Dong-Jin Qian, Li Zhang, Minghua Liu

**Affiliations:** 1 Department of Chemistry, Fudan University, 220 Handan Road, Shanghai 200433, China; 2 Beijing National Laboratory for Molecular Science, CAS Key Laboratory of Colloid, Interface and Chemical Thermodynamics, Institute of Chemistry, Chinese Academy of Sciences, No. 2 Zhongguancun Beiyijie, Beijing 100190, China

**Keywords:** Metal-organic complex nanocrystal, Liquid-liquid interface, Morphology, Fluorescence, Electrochemistry

## Abstract

Immiscible liquid-liquid interfaces provide unique double phase regions for the design and construction of nanoscale materials. Here, we reported Ag(I)-directed growth of metal-organic complex nanocrystals by using AgNO_3_ as a connector in the aqueous solution and bidentate ligand of 1,4-bis(9-O-dihydroquininyl)anthraquinone [(DHQ)_2_AQN] and its enantiomer of (DHQD)_2_AQN in the chloroform solutions as linkers. The Ag-(DHQ)_2_AQN and Ag-(DHQD)_2_AQN complex nanocrystals were formed at the liquid-liquid interfaces and characterized by using UV-vis absorption and fluorescence spectroscopy and X-ray photoelectron spectroscopy, as well as by using scanning electron microscopy. Screw-like nanocrystals were formed at the initial 30 min after the interfacial coordination reaction started, then they grew into nanorods after several days, and finally became cubic microcrystals after 2 weeks. The pure ligand showed two emission bands centered at about 363 and 522 nm in the methanol solution, the second one of which was quenched and shifted to about 470 nm in the Ag-complex nanocrystals. Two couples of reversible redox waves were recorded for the Ag-complex nanocrystals; one centered at about -0.25 V (vs. Ag/AgCl) was designated to one electron transfer process of Ag - (DHQ)_2_AQN and Ag - (DHQ)_2_AQN^+^, and the other one centered at about 0.2 V was designated to one electron transfer process of Ag - (DHQ)_2_AQN and Ag^+^ - (DHQ)_2_AQN.

## Background

Self-assembly of nanostructural materials at the fluid interfaces has recently received growing attention because the interface regions have a double phase thickness of tens of nanometers depending on the nature of the solvents and species within them, a dimension of which is comparable to that of the nanostructural materials [1]. That is, the fluid interface provides a unique region for the growth of micro- or nanoscale materials and for the constrained chemical reactions [2,3]. It has been further found that those materials produced at the fluid interfaces are highly mobile and can rapidly achieve an equilibrium assembly with the reactants in each phase. The dynamic process of species or particles across interface usually dominate composition, morphology, and structure of the materials produced [4,5]. The rapid diffusion of nanoparticles and reagents in either fluid phase leads to very efficient interfacial chemistry, including interfacial chemical reactions and molecular assembly.

Immiscible solutions are often used to form the fluid interface since such an interface can provide a defect-free junction that has an importance for the products with high purity [6]. Many one-dimensional (1D) nanowires and nanotubes and 2D nanosheets and nanocombs of metals, metal oxide, metal sulfide, and complexes have been designed and constructed in the past decades [7-9]. Our previous work has revealed that the specific features of the metal ions (such as the geometry) and coordination numbers of the anionic ions or ligands take an important role in governing the crystal structure of the products [10-12], though a complex interplay of van der Waals, electrostatic, magnetic, molecular, and entropic effects needs to be considered. Besides the inorganic compounds, polymer crystalline nanomaterials could also be constructed at the liquid/liquid interfaces. For instance, Matsui and coworkers synthesized single crystalline conducting polymer, poly(3,4-ethylenedioxythiophene), with the fast conductance switching property [13]. They further prepared single crystalline nanoneedles of polyaniline and polypyrrole via an interfacial polymerization induced by FeCl_3_[14]; the products have a fast conductance switching time between the insulating and conducting states in the order of milliseconds.

Layered porous poly(4-vinylpyridine) (P4VP) films could also be formed at the water-oil interfaces with the porous diameters in the range from hundred nanometers to several micrometers [15]. Liu and coworkers have further developed this method to prepare microcapsules and foam films, which were used as platforms to form various composite inorganic nanomaterials. Examples included gold nanoparticle-doped poly(2-vinylpyridine) and poly(N-vinylcarbazole) composites [16,17] and silver- or gold-doped diblock copolymer of poly(t-butyl methacrylate)-block-poly(2-vinyl pyridine) composites [18]. These metal-doped composites showed high catalytic activity and durability on the reduction of organic compounds such as nitrobenzene, 4-nitrophenol, and 4-nitrobenzoic acid [19].

In the present work, Ag(I)-directed metal-organic complex nanocrystals were fabricated at the water-chloroform interface by using AgNO_3_ as a connector and bidentate chiral ligand of 1,4-bis(9-O-dihydroquininyl)anthraquinone [(DHQ)_2_AQN] and its enantiomer of (DHQD)_2_AQN as linkers. Our previous work has revealed that AgNO_3_ could direct formation of chiral coordination polymers (CPs) at the air-water interface [20]. Atomic force microscopic (AFM) images for the transferred CPs revealed irregular aggregates that were composed of many round particles. These particles were connected together to form wires with particular orientation. However, due to the limitation of the reaction species within the Langmuir monolayers at the air-water interface, the orientation of those aggregates was not clear. Hence, to clarify morphologies of these aggregates, the coordination reaction was performed here at the water-chloroform interface wherein the irregular aggregates may grow into relatively larger macro-/nanocrystals since there were enough inorganic metal ions and ligands in each phase. The as-prepared Ag-organic complex nanocrystals were characterized by using UV-vis absorption spectroscopy and X-ray photoelectron spectroscopy (XPS) and scanning electron microscopy (SEM). Finally, the luminescent behaviors and electrochemical properties of the nanocrystals were investigated.

## Methods

### Materials

**Figure 1:**
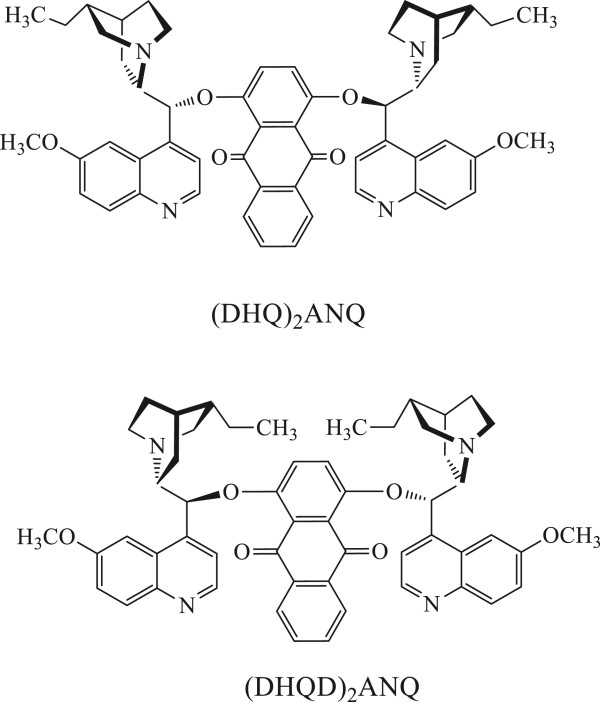
Structure of the ligands used in the present work.

Chiral ligand of hydroquinine anthraquinone-1,4-diyl diether of (DHQ)_2_AQN and its enantiomer (DHQD)_2_AQN (Figure 1) were purchased from Sigma-Aldrich Co. (St. Louis, MO, USA). Chloroform was from Alfa Aesar® (Beijing, China). AgNO_3_ was from Shanghai Chemical Reagent Co. (Shanghai, China). All chemicals were used as received without further purification. Ultrapure water (18.2 ΩM cm) was prepared with a RephiLe filtration unit (RephiLe Bioscience Ltd, Shanghai, China).

### Growth of Ag-complex nanocrystals at the water-chloroform interface

Interfacial self-assembly of the Ag-(DHQ)_2_AQN or Ag-(DHQD)_2_AQN complex nanocrystals was performed as follows: 20 mL 10 mM AgNO_3_ aqueous solution was slowly added onto the surface of 30 mL (DHQ)_2_AQN or (DHQD)_2_AQN chloroform solution in a beaker. The reaction system was left undisturbed at room temperature from several minutes to 2 weeks. As a control experiment, interfacial phenomenon of (DHQ)_2_AQN or (DHQD)_2_AQN at the interface of pure water and ligand chloroform solution was also investigated.

### Transfer of Ag-complex nanocrystals onto substrate surfaces

Layers of Ag-(DHQ)_2_AQN or Ag-(DHQD)_2_AQN complex nanocrystals grown at the interface were transferred onto substrate surfaces with the use of a dipper from KSV 5000 minitrough (KSV Instrument Co., Helsinki, Finland) or from JML04C2 trough (Powereach, Shanghai, China). The substrate was firstly immersed into the liquid-liquid interface before the interfacial reaction started, and after a given time waiting for the coordination reaction and formation of Ag-complex nanocrystals, the substrate was vertically withdrawn from the interface. The dipping rate was kept at 1 mm/min.

### Instruments

UV-vis spectra were measured with the use of a Shimadzu UV-2550 UV-vis spectrophotometer (Shimadzu, Kyoto, Japan). Steady-state fluorescence spectra were recorded by using a Shimadzu RF-5300PC spectrophotometer.

XPS spectra were recorded by using a VG ESCALAB MKII multifunction spectrometer (VG Scientific, East Grinstead, UK), with nonmonochromatized Mg-Kα X-rays as the excitation source. The system was carefully calibrated by Fermi edge of nickel, Au 4f_2/7_, and Cu 2p_2/3_ binding energy. Pass energy of 70 eV and step size of 1 eV were chosen when taking spectra. In the analysis, chamber pressures of 1 ~ 2 × 10^-7^ Pa were routinely maintained. The binding energies obtained in the XPS analysis were corrected by referencing the C1s peak to 284.60 eV.

Scanning electron microscopic (SEM) measurements were performed on a Philips XL30 electron microscope (Philips, Amsterdam, The Netherlands). The samples were deposited on the Si substrate surface. High-resolution transmission electron microscope image was acquired on a JEOL JEM-2010 transmission electron microscope (JEOL Ltd., Akishima-shi, Japan) operating at an accelerating voltage of 200 kV. The sample was deposited onto a 230-mesh copper grid covered with Formvar.

Cyclic voltammogram (CV) was measured by using an electrochemical analyzer (CHI 601b, CH Instruments, Inc., Shanghai, China). A Pt wire and Ag/AgCl electrode were used as the auxiliary and reference electrodes, respectively, and the indium tin oxide (ITO) electrode covered with layers of Ag-(DHQ)_2_AQN or Ag-(DHQD)_2_AQN nanocrystals was used as the working electrode with a 10 mmol/L HClO_4_ solution as the electrolyte. For the CV measurement of the ligand redox reaction, an initial potential of -0.5 V was applied for 2 s, followed by cyclic scans to a final potential of 0 V. For the measurement of the Ag(I) redox reaction, the initial potential of -0.1 V was applied for 2 s, followed by cyclic scans to the final potential of 0.5 V. All CV measurements were done for 10 cycles under an Ar atmosphere at room temperature.

## Results and discussion

### Growth of Ag-complex nanocrystals at the water-chloroform interface

Interfacial reaction between the silver ion and ligand of (DHQ)_2_ANQ or (DHQD)_2_ANQ occurred quickly. During experiments, we found that the reaction rate was closely dependent on the concentration of AgNO_3_ in water and that of the ligand in chloroform as well as the temperature. Similar to those reported in the literature [21], stronger concentration of the reactants and higher reaction temperature could result in a quick formation of metal-organic complex nanocrystals. These nanocrystals were then transferred on various substrate surfaces by the vertical dipping method for the characterization of morphologies, absorption and emission spectra, XPS, and electrochemistry.

### Morphology characterization

**Figure 2:**
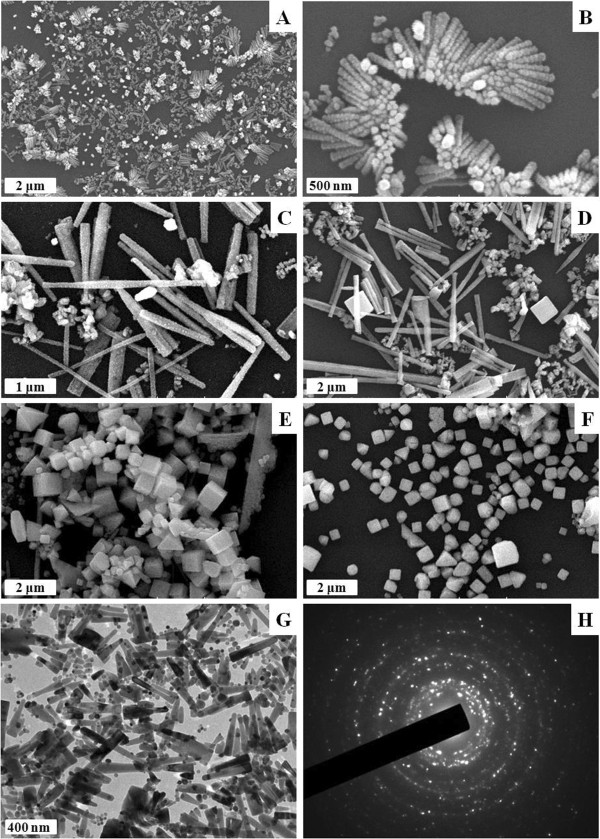
**SEM and TEM images of Ag-(DHQ)**_**2**_**AQN nanocrystals grown at water-chloroform interface after different reaction times.** SEM images after **(A)** 30 min, **(B)** 30 min (enlarged photo), **(C)** 2 h, **(D)** 12 h, **(E)** 3 days, and **(F)** 15 days; **(G)** TEM image after 2 h; **(H)** electron diffraction pattern of the nanocrystals.

Morphologies of the Ag-directed complex nanocrystals were characterized by using SEM technique. These nanocrystals were deposited on the freshly cleaned Si substrate surface by vertical dipping method. Similar morphologies were obtained for the two ligands, so as an example, Figure 2 shows several SEM images of the Ag-(DHQ)_2_AQN complex nanocrystals formed at the water-chloroform interface from the initial 5 min to 2 weeks, which revealed the following features.

Firstly, many screw-like nanocrystals were formed at the initial 30 min with a length of several hundred nanometers and diameters of tens of nanometers (Figure 2A,B). These nanocrystals formed aggregates possibly due to a strong interaction between each screw-like nanocrystal. Such kind of interactions between adjacent nanocrystals has been used to control the growth of large-scale or colloidal nanocrystal building blocks in the organic solutions [22]. As it has been reported that each silver ion may coordinate with two pyridyl groups [20,23], so we may suggest that each silver ion coordinated with two ligands to form Ag-directed CP nanocrystals. The screw-like feature of the nanocrystals may be due to the fact that the ligand was a chiral molecule, which dominated generally the formation of unique supramolecular aggregates or nanocrystals as having been reported by several research groups [24-26].

Secondly, the screw-like nanocrystals transformed into nanorods after the interfacial reaction time increased to about 30 min. As shown in Figure 2C,D, the length of nanorods increased to be about several micrometers with the diameters about tens of nanometers, a little increased as compared with those of the screw-like nanocrystals. This increase may be attributed to the following reasons: (1) the coordination reaction of the silver ions and bidentate ligands continued on the surface of the screw-like nanocrystals, and (2) the nanocrystals formed at the initial time were of highly active surface energy that resulted in a strong interaction between each nanocrystal. As a result, the small nanocrystals formed larger particles as those often occurred in the air-organic solvent interfaces [27].

Thirdly, when the interfacial reaction continued up to several days, the nanorods further grew into cubic macrocrystals with the length of a side about hundreds of nanometers (Figure 2E,F). This process was similar to that we have observed for metal-mediated nanocrystals of multiporphyrin arrays [10], wherein the shapes of the products were closely dependent on the geometries of the central metal ions. Here, the Ag^+^ ions were tetrahedrally coordinated with bidentate ligand of (DHQD)_2_AQN, so screw-like or nanorods were firstly formed, then they grew into larger cubic particles. A comparison of the size of the nanorods with that of the cubic particles could further find that the cubic length was shorter than that of the nanorods; this phenomenon may be attributed to a slow kinetic process of the crystal growing at the earlier stage, then to a thermodynamic process after several days. The other possible reason may be the lowest surface active energy of the cubic crystals; that is, the nanocubes may be more stable than the nanorods.

Similar TEM images were observed for the Ag-(DHQ)_2_AQN complex nanocrystals, but the screw-like nanocrystals formed at the initial time were not very stable under the high beam energy of TEM. As an example, Figure 2G shows a TEM photo of the Ag-complex nanocrystals at the reaction time of 2 h, which revealed that it was composed of many nanorods and dot-like aggregates and in agreement with that observed from the SEM photos. Electron diffraction pattern of the present Ag-complex nanocrystals revealed many irregular dots (Figure 2H), indicating that they were polycrystalline.

### X-ray photoelectron spectroscopy

**Figure 3:**
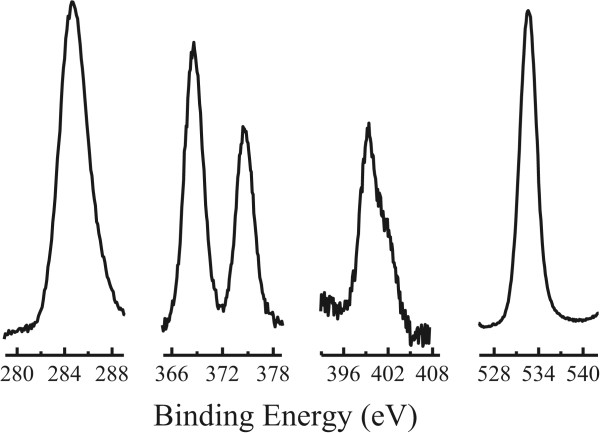
**High-resolution XPS spectra of the Ag-(DHQ)**_**2**_**AQN nanocrystals.**

Element compositions for the Ag-directed complex nanocrystals were detected by using the XPS technique. Also as an example, Figure 3 shows the high-resolution XPS bands for the Ag-(DHQ)_2_AQN complex nanocrystals, which revealed four peaks in the binding energy from 100 to 600 eV except for the Si element from substrate surface. The binding energy of these four peaks was as follows: 284.6, 368.6/374.4, 399.4 ~ 403, and 532.6 eV, which could be assigned to the elements of C(1 s), Ag(3d), N(1 s), and O(1 s), respectively. The C, part of N, and O elements were from the ligand of (DHQ)_2_AQN, while the elements of Ag, part of N, and O were from AgNO_3_. Thus, these XPS data confirmed formation of Ag-(DHQ)_2_AQN complex nanocrystals [20].

### Absorbance and fluorescence emission of the nanocrystals

**Figure 4:**
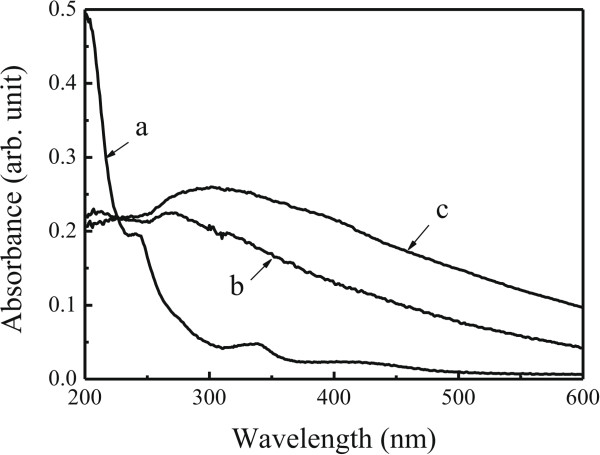
**Absorption spectra.** (a) Ligand of (DHQ)_2_AQN in the methanol solution and (b) Ag-(DHQ)_2_AQN nanocrystals deposited 2 h and (c) Ag-(DHQ)_2_AQN nanocrystals deposited 3 days after the interfacial reaction.

Absorption and emission features of the Ag-complex nanocrystals as-prepared were investigated on the quartz surfaces. As an example, Figure 4 shows absorption spectra for the Ag-(DHQ)_2_AQN nanocrystals transferred from the water-chloroform interface at different reaction times, together with a spectrum of the ligand in the dilute methanol solution. Three absorption bands were recorded and appeared at about 230 ~ 238, 323 ~ 334, and 416 nm for the ligand of (DHQ)_2_AQN in the solution, which can be designated to the electron transition of quinuclidine, quinine, and anthraquinone substituents. Our previous work has revealed that these peaks shifted to about 240, 337, and 416 nm in its Langmuir-Blodgett (LB) film [20]. When the ligand was coordinated with Ag^+^ ions to form the LB film of Ag-(DHQ)_2_AQN coordination polymers, these absorption bands appeared at about 245, 342, and 413 nm, respectively [20]. That is, a redshift was recorded for the former two peaks when the ligand was coordinated with Ag^+^ ions.

Here, for the films of Ag-(DHQ)_2_AQN complex nanocrystals, a broad absorption band was recorded with the maximum at about 270 ~ 296 nm. But it is hard to distinguish each peak as those in the solutions and LB films [20]. This difference can be attributed to the fact that the LB film was almost transparent and did not scatter the light during the absorption measurements; however, the present film of Ag-(DHQ)_2_AQN complex nanocrystals was not. The detected light was strongly scattered by the nanoparticles, resulting in a broaden band from 200 to nearly 600 nm. Moreover, with increasing the reaction time, the average sizes of the nanocrystals increased (as shown in the SEM images in Figure 2). As a result, the main absorption band a little red shifted, the feature of which was in agreement with that observed in the aggregates of inorganic complexes or macrocyclic compounds due to a strong molecular interaction in the larger aggregates or to a stronger light scattered by the larger aggregates [28,29].

Based on the chemical structure of the chiral ligands used, we can find that they contain both quinine and anthraquinone substituents, both of them are important light-harvesting units. They can not only absorb ultraviolet light but also give off emission in the near ultraviolet and visible region [30,31], so they have potential applications in the fields of optical, electroluminescent materials and light-emitting diodes. The light energy absorbed by the ligands can be further transferred to some metal ions like Eu^3+^ and Tb^3+^ as luminophores, sensors, and organic light-emitting diodes [32,33]. Here, luminescent emission properties for the Ag-(DHQ)_2_AQN complex nanocrystals were investigated after they were transferred on the quartz substrate surfaces.

**Figure 5:**
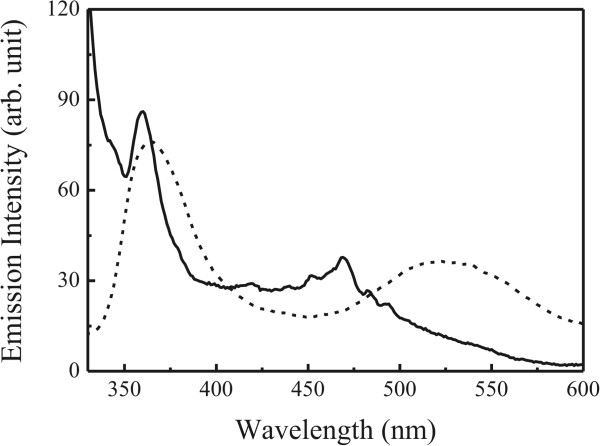
**Fluorescence spectra.** Ag-(DHQ)_2_AQN nanocrystals (solid line) and the ligand in the methanol solution (dashed line).

Figure 5 shows emission spectrum for the Ag-(DHQ)_2_AQN complex nanocrystals on the quartz substrate surface, together with an emission spectrum of the ligand in the methanol solution. The excited wavelength was 317 nm. These emission spectra revealed the following features. Firstly, two broad emission peaks were recorded and centered at about 363 and 520 to 530 nm for the ligand (DHQ)_2_AQN in the methanol solution. The first peak may be designated to the emission from the hydroquinine substituents and the second one to that of the anthraquinone [20]. Secondly, the Ag-(DHQ)_2_AQN complex nanocrystals showed also two broad emission peaks; the first one appeared at about 360 nm (very similar to that in the methanol solution), while the second one ‘blue’ shifted to about 470 nm. Previously, we have found that the fluorescent emission features for the ligands in the casting films were similar to those in the LB films; that is, the first one appeared at about 365 nm while the second one slightly red shifted to the range of 530 ~ 560 nm. This redshift has been attributed to a closely packed arrangement of the molecules in the organized ultrathin films as often observed for the macrocyclic molecules such as porphyrins and inorganic complexes [10-12].

**Figure 6:**
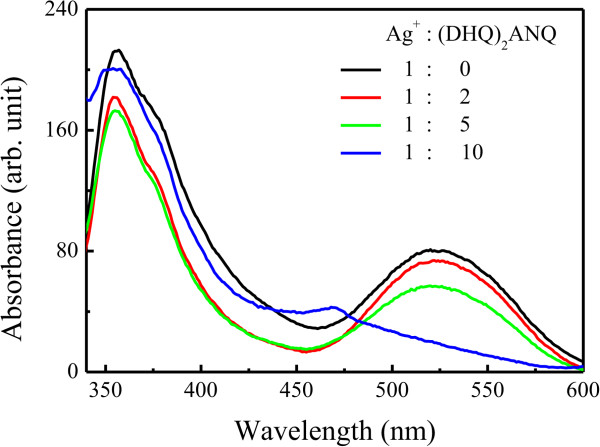
**Fluorescence spectra for the mixtures of (DHQ)**_**2**_**AQN and AgNO3 in the methanol solutions.**

The blueshift phenomenon of the second emission band was also observed in the layer-by-layer multilayers of Pd-(DHQ)_2_ANQ and Pd-(DHQD)_2_ANQ coordination polymers [34], which was attributed to the formation of the Pd-(DHQ)_2_AQN and Pd-(DHQD)_2_ANQ complexes. During experiments, we measured the emission spectra for the mixtures of (DHQ)_2_ANQ and AgNO_3_ at the molar ratios from 1:0 to 1:10 in the methanol solution. As shown in Figure 6, the emission at approximately 360 nm did not show a significant difference with the increase of the relative molar fractions of AgNO_3_. However, the emission at 525 nm gradually weakened when the AgNO_3_ solution was added. When the molar ratios of AgNO_3_ relative to the ligand increased to 10, a weak emission peak was observed at approximately 470 nm, which was in agreement with that observed in the Ag-(DHQ)_2_AQN complex nanocrystals. These results suggested that the blueshift and quenching of the second emission peak may be attributed to the formation of metal-ligand complexes in the nanocrystals [34].

### Voltammetric properties

Besides the interesting optical and chiral behaviors, (DHQ)_2_AQN and (DHQD)_2_AQN are also electroactive compounds because they contain the anthraquinone substituents, which have been widely used as electroactive materials either for the fundamental researches on electrochemistry or for the potential applications in the sensors, electrochromism, and organic batteries [32,33]. Here, the cyclic voltammograms of Ag-complex nanocrystals on the ITO electrodes were investigated and compared with those of the ligand in the casting films.

**Figure 7:**
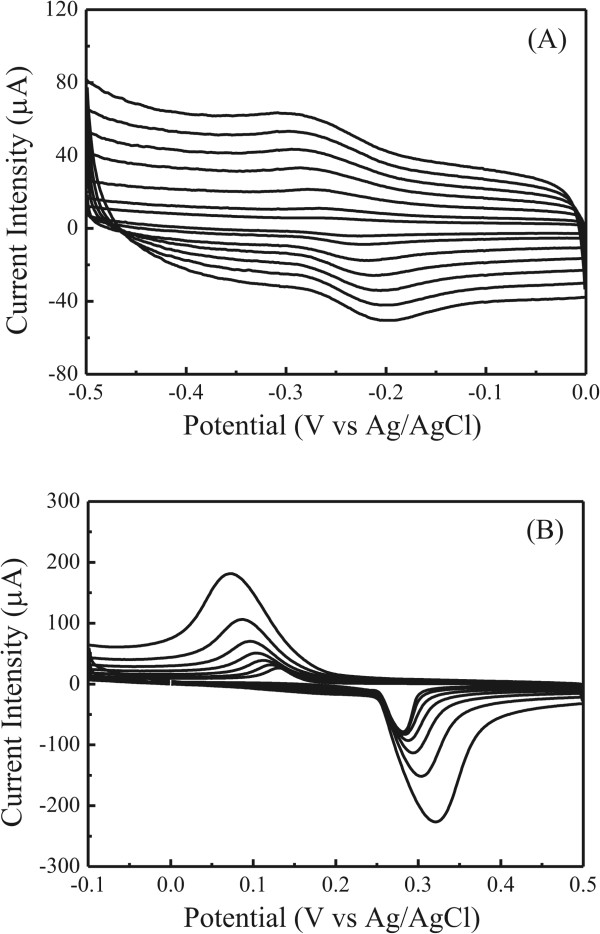
**Cyclic voltammograms of the Ag-(DHQ)**_**2**_**AQN nanocrystals. (A)** Potential range of -0.5 to 0 V and **(B)** potential range of -0.1 to 0.5 V at the scan rates of 0.05, 0.1, 0.2, 0.3, 0.4, 0.5, and 0.6 V/s in the 0.01 mol/L HClO_4_ electrolyte solutions.

Figure 7A shows the CV curves for the ITO electrode covered by the Ag-(DHQ)_2_AQN complex nanocrystals in the 0.01 mol/L HClO_4_ electrolyte solutions in the potential range of -0.5 to 0 V at the scan rates from 0.05 to 0.6 V/s. One couple of redox wave was recorded with the cathodic (*E*_pc_) and anodic (*E*_pa_) potentials at around -0.27 ~ -0.29 and -0.22 ~ -0.20 V vs. Ag/AgCl, respectively. Based on the literature [35], this redox couple was designated to the electron transfer process of (DHQ)_2_AQN and (DHQ)_2_AQN^-^. The potential difference Δ*E* (Δ*E* = *E*_pa_ – *E*_pc_) was 0.05 V when the scan rate was 0.05 V/s, which slightly increased to 0.09 V when the scan rate was 0.6 V/s. The reduction current intensity was about 15.6 μA, which was also close to that of the oxidation current intensity (12.6 μA) when the scan rate was 0.6 V/s. These CV features suggested that the redox process of the ligands in the nanocrystals was reversible.

Figure 7B shows the CV curves for the Ag-(DHQ)_2_AQN nanocrystals in the 0.01 mol/L HClO_4_ electrolyte solutions in the potential range of -0.1 to 0.5 V at the scan rates from 0.05 to 0.6 V/s. One couple of redox wave was recorded with the cathodic and anodic potentials at around -0.13 ~ 0.07 and 0.28 ~ 0.32 V, which was attributed to the redox reaction of the connector of Ag^+^ ions [36], with the electron transfer process of Ag-(DHQ)_2_AQN and Ag^+^-(DHQ)_2_AQN. The potential difference Δ*E* was 0.35 V when the scan rate was 0.05 V/s, which increased to 0.45 V when the scan rate was 0.6 V/s.

**Figure 8:**
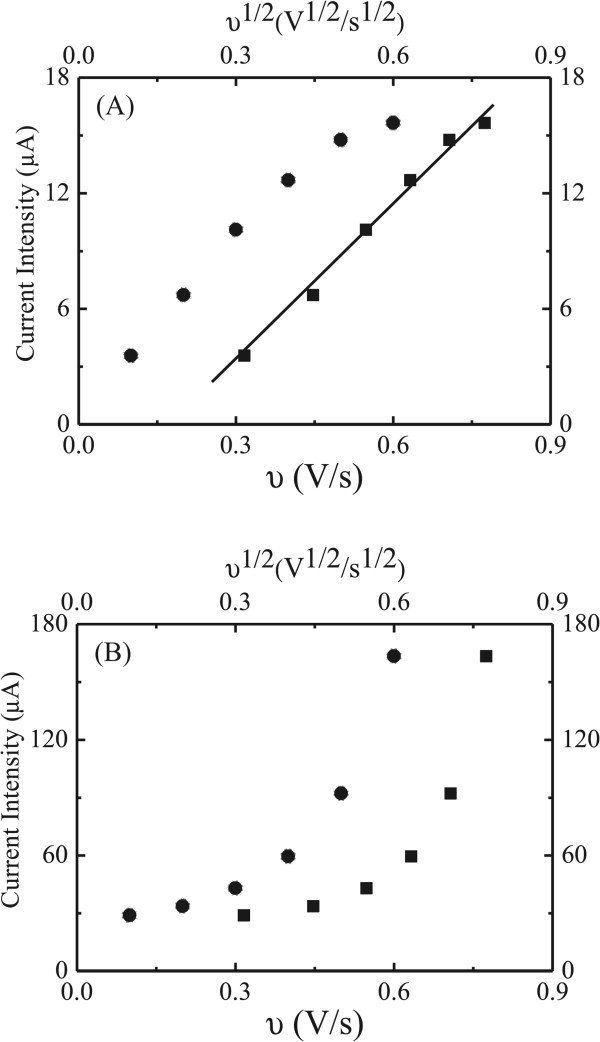
**Plots of current intensity to the scan rates (black circles) and root of scan rates (black squares). (A)** Reduction reaction of Ag-(DHQ)_2_AQN → Ag-(DHQ)_2_AQN^-^ and **(B)** reduction reaction of Ag^+^-(DHQ)_2_AQN → Ag-(DHQ)_2_AQN complex nanocrystals.

Relation of the redox current intensity of the modified electrode to the scan rate and the root of the scan rate was calculated. Figure 8A shows plots of the current intensity for the reduction reaction of the ligand Ag-(DHQ)_2_AQN → Ag-(DHQ)_2_AQN^-^ to the scan rate and the root of the scan rate for the ITO electrode modified by the film of Ag-(DHQ)_2_AQN nanocrystals. Based on these data, we can find that the current intensity was proportional to the root of the scan rates rather than that of the scan rates, which indicated that the electroactive thickness of the nanocrystals was thicker than that of the diffusion layer. This was reasonable because the film was composed of irregular Ag-(DHQ)_2_AQN complex nanocrystals with the sizes in the range of hundreds of nanometers (Figure 2). A close inspection of the figure could further find that this line did not go through the zero point. This feature suggested that, besides the diffusion layer, the electron transfer process between the Ag-(DHQ)_2_AQN nanocrystals and electrode surface may be also influenced by some other issues, such as the interfacial resistance between the nanocrystals and electrode surface and the connectors of AgNO_3_ between the ligands and electrode surface [36].

Figure 8B shows plots of the current intensity for the reduction reaction of the silver ions in the complex nanocrystals of Ag^+^-(DHQ)_2_AQN → Ag-(DHQ)_2_AQN to the scan rate and the root of the scan rate. Different from that observed in Figure 8A for the reduction reaction of the ligands, here, the current intensity was not proportional to neither the scan rate nor the root of the scan rate. According to the theory of film-modified electrode [37,38], this phenomenon could be attributed to that the electroactive thickness of the nanocrystals was not very thinner or very thicker as compared with that of the diffusion layer.

## Conclusions

Silver(I)-directed metal-organic complex nanocrystals have been prepared using AgNO_3_ as a connector and chiral bidentate ligands as linkers at the water-chloroform interface. Screw-like Ag-directed complex nanocrystals formed at the initial reaction time; then, they grew into nanorods and finally became cubic nano- or microcrystals. The as-prepared Ag-complex nanocrystals showed strong luminescent emissions as well as reversible redox properties, which may have potential interests in the fundamental researches and applications in the fields of chemically modified electrodes and optoelectronic devices.

## Competing interests

The authors declare that they have no competing interests.

## Authors’ contributions

YT, HTW, MC, and LZ carried out the synthesis and characterizations of the materials and drafted the manuscript. ML and DJQ contributed in the design and discussion of this work and in the revision of the manuscript. All authors read and approved the final manuscript.
